# MicroRNA-107 promotes proliferation of gastric cancer cells by targeting cyclin dependent kinase 8

**DOI:** 10.1186/s13000-014-0164-1

**Published:** 2014-08-28

**Authors:** Yan-qiang Song, Xu-hui Ma, Gui-liang Ma, Bin Lin, Chao Liu, Quan-jiang Deng, Wen-ping Lv

**Affiliations:** Department of general surgery, Qingdao Municipal Hospital (East), Medical College of Qingdao University, No.5 Donghai Middle Road, Qingdao, 266071 People’s Republic of China

**Keywords:** MiRNA-107, Proliferation, CDK8, Gastric cancer

## Abstract

**Background:**

The biological processes and molecular mechanisms underlying miR-107 remain unclear in gastric cancer(GC). In this study, we aimed to investigate the expression, biological functions and mechanisms of miR-107 in GC.

**Methods:**

Quantitative real-time RT-PCR was used to test miR-107 expression. MTT and colony formation assays were conducted to explore the potential function of miR-107 in human GC cell line SGC7901. The target gene was determined by bioinformatic algorithms, dual luciferase reporter assay, RT-PCR and Western blot.

**Results:**

Expression of miR-107 was significantly elevated in GC cell line than that in gastric epithelial cell line(p = 0.012). We found that miR-107 inhibitor transfection significantly decreased the proliferation of GC cell line, and clone formation rate of miR-107 inhibitor transfected group was significantly lower than that of control group. Luciferase assays using a reporter carrying a putative miR-107 target site in the 3′untranslated region (3′-UTR) of cyclin dependent kinase 8 (CDK8) revealed that miR-107 directly targets CDK8. The expression level of CDK8 mRNA and protein in miR-107 inhibitor transfected GC cell line was significantly decreased compared with control group.

**Conclusion:**

Our findings indicate that miR-107 is upregulated in GC and affects the proliferation of GC cells, partially through the regulation of CDK8.

**Virtual Slides:**

The virtual slide(s) for this article can be found here: http://www.diagnosticpathology.diagnomx.eu/vs/13000_2014_164

## Background

Gastric cancer (GC) is the fourth most common malignancy and the second most common cause of death from cancer in the world [1]. In general, GC has a 5‑year overall survival rate of ~15% and for patients with advanced GC, the median overall survival is <1 year [2]. Considering these statistics, increased research into potential preventative methods for GC is required, as well as improved early detection and more effective treatments.

MicroRNAs (miRNAs) are a recently discovered class of small (approximately 18–24 nucleotides in length), noncoding regulatory RNAs that negatively regulate gene expression at the posttranscriptional and/or translational level. miRNAs can trigger cleavage of target mRNAs or inhibit protein translation through sequence-specific interactions with the 3′-untranslated regions (3′-UTRs) of the target mRNAs [3–5]. Although the full extent of the biological functionalities of miRNAs has yet to be identified, they have been suggested to act as intrinsic regulators of many cellular processes including cell invasion, differentiation, proliferation, and apoptosis [6,7]. Furthermore, aberrant expression of miRNAs has been linked to the development and progression of cancer and has been shown to have prognostic significance [8]. Accumulating evidence shows that microRNA-107(miR-107) is one of the oncogenic RNAs, and overexpression of these RNAs has been reported in several types of human malignant solid tumors, including gastric, esophageal, pancreatic and colorectal cancer [9–12]. Previously, Inoue et al. found that the mean expression level of miR-107 was significantly higher in the GC tissues compared to that of normal tissues. In the comparison of clinicopathological factors, miR-107 expression showed significant association with depth of tumor invasion, lymph node metastasis and tumor stage [13]. However, the detailed mechanisms of miR-107 were fewly investigated in GC.

Cyclin dependent kinase 8 (CDK8) locating on chromosome13q12 has five transcripts and only one transcript encodes protein product containing 464 amino acid residues (molecular weight 53.2 kD). CDK8 has important function on the regulation of gene transcription [14]. Recent studies suggest that CDK8 is important in the process of tumor development [15]. CDK8 plays a key role in the regulation of cell cycle and cell growth on post-transcriptional level, and promotes the development and progression of colorectal cancer [16]. We found that miR-107 might modulate CDK8 using online prediction software Target Scan. In our study, we sought to investigate the crucial role of miR-107 in GC. We identified that miR-107 could regulate proliferation of GC by targeting CDK8.

## Methods

### Cell lines

Human GC cell line SGC7901 and a non-malignant gastric epithelium cell line GES-1 were obtained from the Chinese Academy of Sciences (Shanghai, China), and cultured in RPMI-1640 medium with 10% FBS. HEK-293 cell was cultured in Dulbecco’s modified Eagle’s medium with 10% FBS. All cell lines were incubated at 37°C with 5% CO2.

### Total RNA extraction and qRT-PCR

Total RNA was isolated by TRIzol Reagent (Invitrogen, CA, USA). SYBR Premix Ex Taq (Takara, Tokyo, Japan) was used to assess gene expression on ABI Stepone plus (ABI, CA, USA). miRNA was isolated with All-in-one microRNA extraction kit (GeneCopoeia, CA, USA). Primers for CDK8: forward, 5′-GCCGGCATAGACGCGTGCTGCATCGGAATC-TTGTC-3′ and reverse, 5′-ATCCTTTATTAAGCTTACCACATACAAAGACAAATGCTT-3′.

Primers for GAPDH: forward, 5′-ACGGATTTGGTCGTATTGGGC-3′, and reverse, 5′-TTGACGGTGCCATGGAATTTG-3′. Primers for miR-107 and U6 were obtained from GeneCopoeia. The expression of CDK8 was normalized with GAPDH, and the expression of miR-107 was normalized with U6.

### MTT assay for proliferation activity

SGC7901 cells was cultured in 25 cm^2^ culture flask to approximately 80%-90% density collected by digestion and centrifugation, and then seeded into 96 well plates at 1,000 cells/well. 96-well plate was placed in cell culture incubator until cell monolayer reaches 40%-50% density. MiR-107 inhibitor was transfected into cells according to Invitrogen Lipofectamine reagent instructions. 20 μl MTT solution (5 mg/ml) was added daily for 4 days. Supernatant was discarded and 150 μl DMSO (dimethyl sulfoxide) was added to each well. Then plates were placed on low-speed shaker for 10 min to fully dissolve the crystals. Absorbance at 490 nm was measured by the multi-plate reader. Experiment was performed with sextuplicates and repeated for 3 times.

### Colony-forming unit assay

miR-107 inhibitor or control was transfected into SGC7901 cells at 100 nM concentrations. 48 h later transfected cells were seeded into 6-well plates as 500 cell/well with triplicate and incubated at 37°C, 5% CO2 for 7 days until visible cloning were observed in the dish. Culture medium was discarded. Each well was washed with PBS twice carefully. Then cells were fixed with 4% paraformaldehyde for 10 min and stained with 0.1% crystal violet. After washed with running water three times and dried at room temperature, each well was observed and photographed. Cell colonies with more than 50 cells were counted under the microscope. Clone formation rate was calculated as following formula: Clone formation rate = number of formed colony/number of seeded cells × 100%.

### miR-107 target gene prediction and 3′-UTR plasmid vectors construction

miRNA target genes were predicted using online prediction software miRanda, TargetScan and PicTar. 3′-UTR region of CDK8 including miR-107 targeting sequence was amplified using PCR amplification. Upstream primer: 5′-GCCGGCATAGACGCGTGCTGCATCGGAATC-TTGTC-3′, downstream primer: 5′-ATCCTTTATTAAGCTTACCACATACAAAGACAAATGCTT-3′ (Mlu I, Hind III restriction sites were underlined). Target sequence after T-A clone was sub-cloned into the vector pMIR and inserted into the downstream of firefly luciferase gene. This recombinant vector was named as pMIR-REPORT. All constructed plasmids were validated by restriction analysis and DNA sequencing.

### Luciferase report gene detection

HEK293 cells in logarithmic growth phase were seeded into 96-well culture plate, incubated at 37°C, 5% CO_2_ for 24 h, and co-transfected with pMIR-REPORT and miR-107 mimics (or NC) using Lipofectamine 2000. Experiment was performed with sextuplicates and repeated for 3 times. Detection was performed following instruction of dual luciferase reporter gene assay kit. Cell culture medium was discarded after 24 h transfection. Wells were washed three times with PBS and treated by 20 μl cell lysis buffer for 15 min at room temperature. 100 μl firefly luciferase detection solution was added to detect firefly luciferase activity, then 100 μl renilla luciferase dection reagent was added to measure renilla luciferase activity. Luciferase activity (C) = firefly luciferase/renilla luciferase activity. Relative luciferase activity was calculated in each group.

### Western blotting

Total protein was extracted from GC cell line. The concentration of proteins was measured by BCA protein assay kit (Pierce, IL, USA). Proteins were separated by 10% SDS-PAGE gel and transferred to polyvinylidene difluoride(PVDF) membrane (Millipore, MA, USA). The membrane was first incubated with specific primary antibodies, then with secondary antibodies labeled with HRP and detected by ECL.

### Statistical methods

Experimental data was showed as mean ± SD. Two groups were compared using t-test comparison and multi-groups were compared using variance analysis by SPSS18.0 statistical software. P < 0.05 indicates significant difference.

## Results

### miR-107 was elevated in GC cell line SGC7901

**Figure 1:**
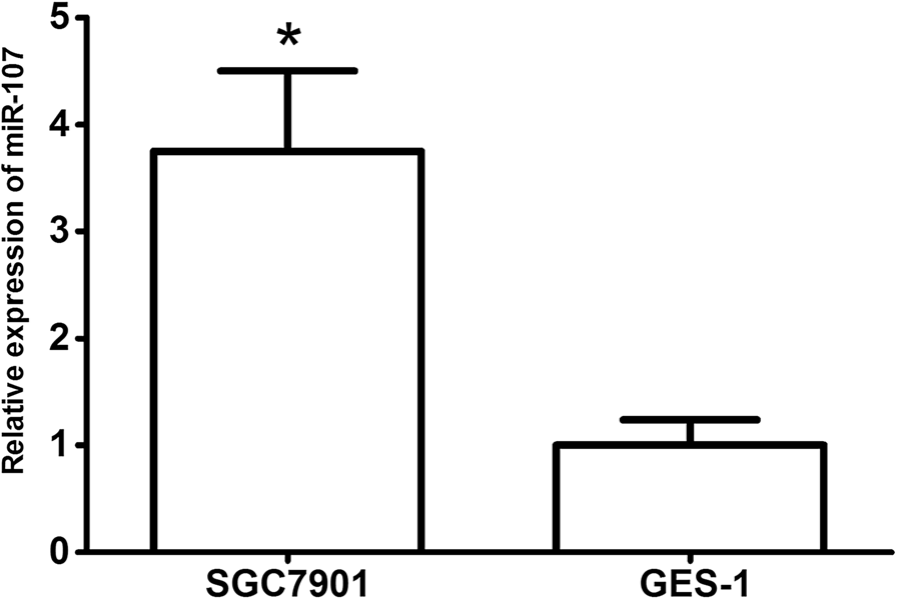
**miR-107 was elevated in GC cell line.** *P < 0.05.

qRT-PCR was used to detect the expression of miR-107 in GC cell line, SGC7901, and a gastric epithelial cell line, GES-1. Expression of miR-107 was significantly elevated in GC cell line, SGC7901 (*P* = 0.012, shown in Figure 1).

### miR-107 promoted GC cell line proliferation

**Figure 2:**
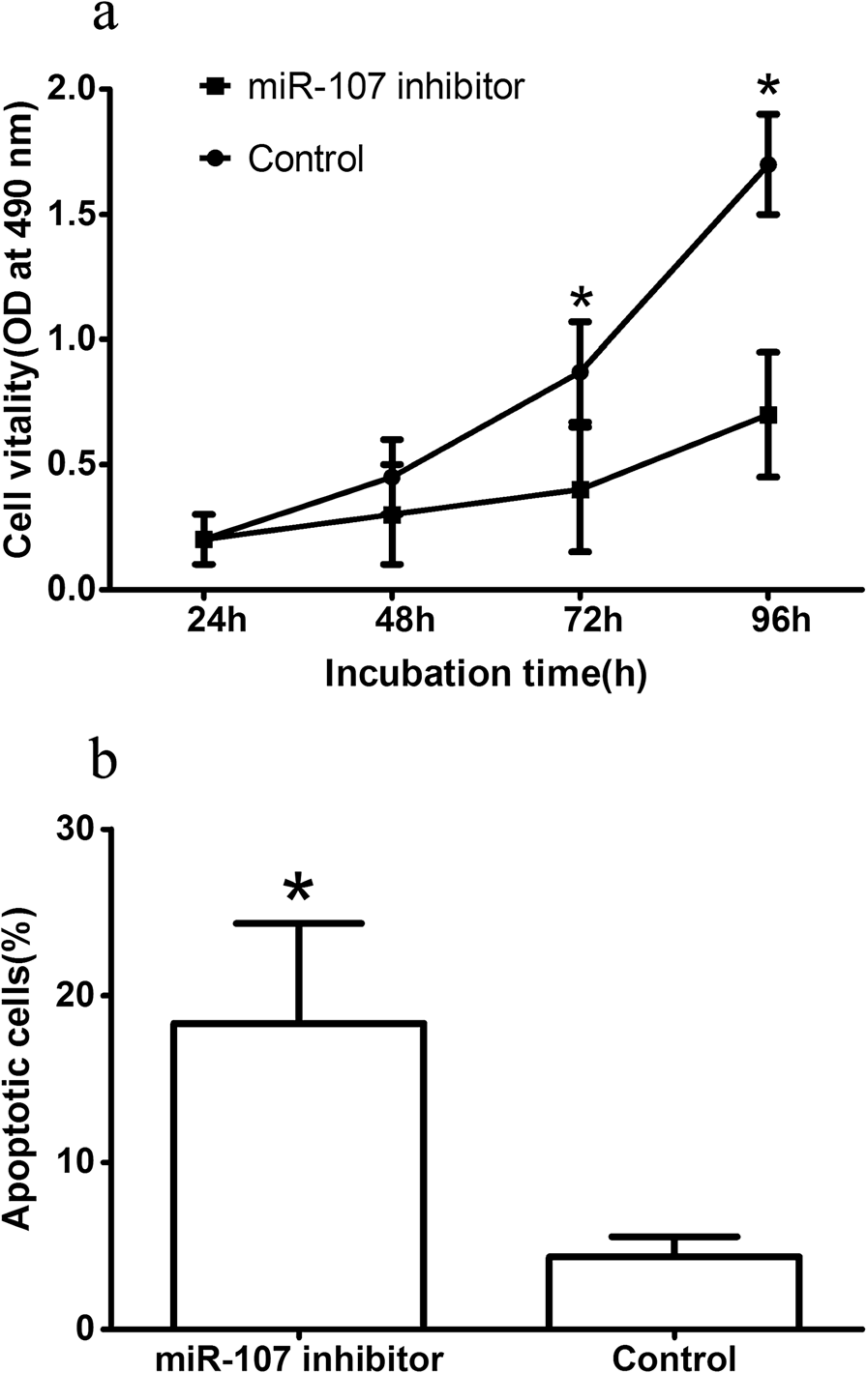
**miR-107 promoted GC cell line proliferation. a** Inhibition of miR-107 significantly decreased cell proliferation in SGC7901 cells. **b** The proportion of apoptotic SGC7901 cells induced by miR-107 inhibitor was significantly greater than that induced by the negative control. *P < 0.05.

We investigated the effect of miR-107 on the proliferation of GC cell line SGC7901. We found that miR-107 inhibitor transfection significantly decreased the proliferation of SGC7901 (shown in Figure 2a). We further explored the effect of miR-107 on apoptosis and found that apoptosis was increased dramatically in SGC7901 cells 72 h after transfection of miR-107 inhibitor (shown in Figure 2b), suggesting that miR-107 might function as an antiapoptotic factor in human GC cells.

### miR-107 inhibitor decreased GC cell line SGC7901 clone formation rate

**Figure 3:**
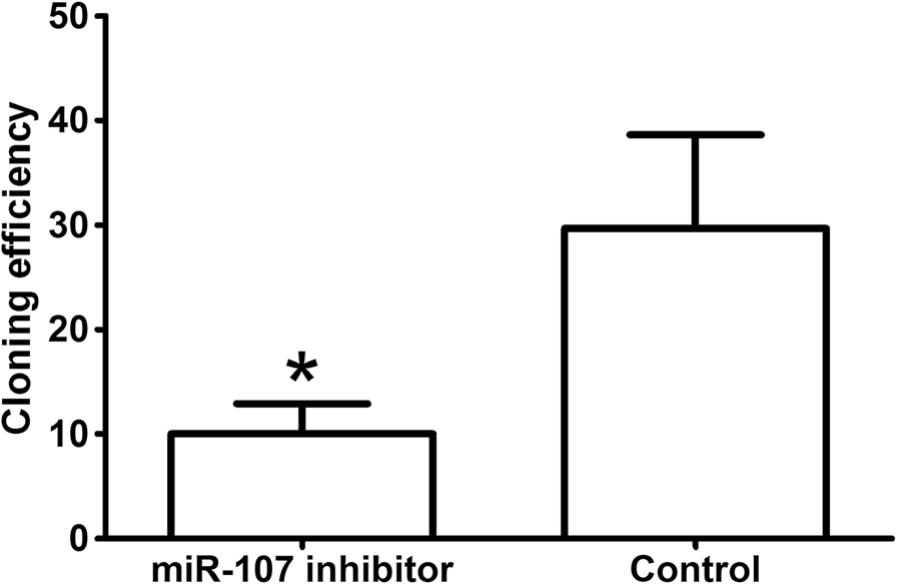
**miR-107 inhibitor inhibited cell colony formation.** *P < 0.05.

Clone formation rate of miR-107 inhibitor transfected group was significantly lower than that of control group, demonstrating that miR-107 inhibitor significantly inhibited GC cell line SGC7901 colony formation (*P* < 0.05, shown in Figure 3).

### CDK8 was a direct target of miR-107

**Figure 4:**
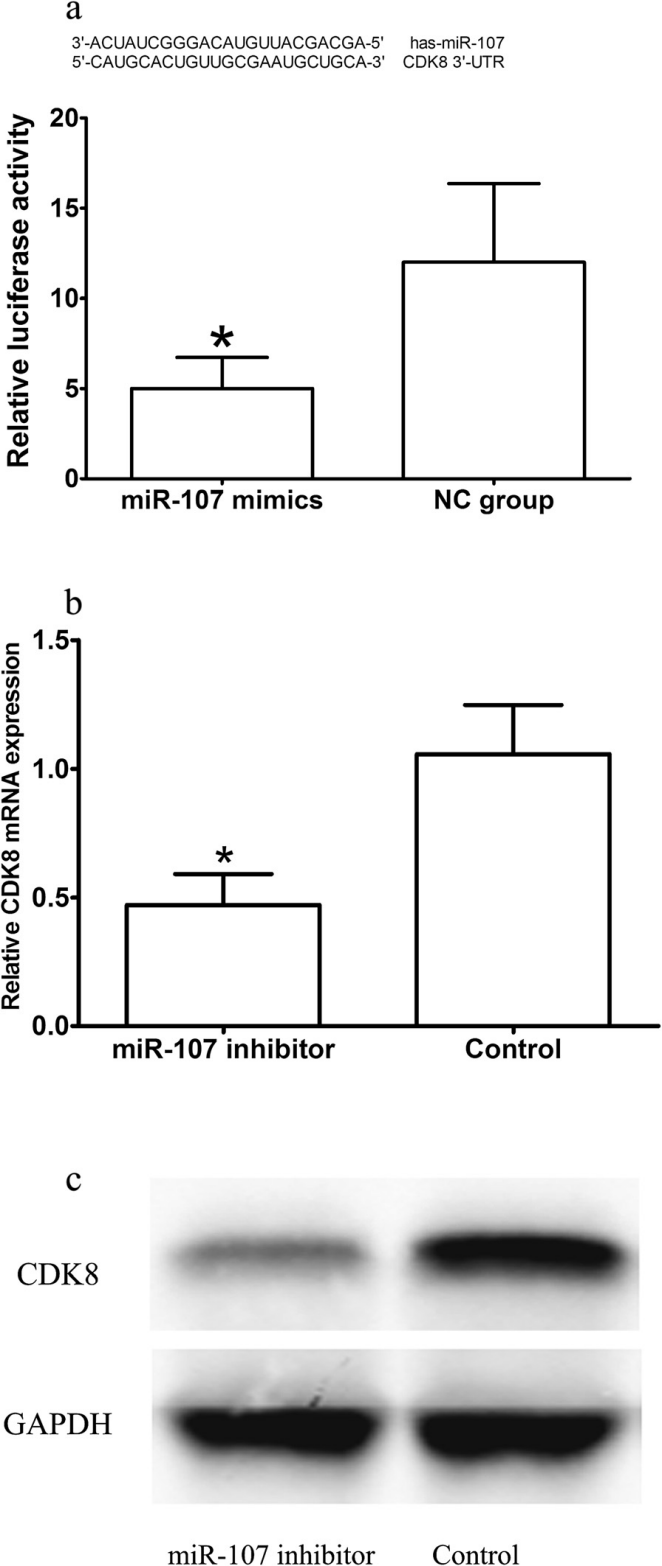
**CDK8 was a direct target of miR-107. a**: The relative luciferase activity (firefly/renilla) was measured in HEK293 cells after cotransfection of the CDK8 luciferase construct with either miR-107 mimics or NC. **b**: CDK8 mRNA level was detected by RT-PCR in SGC7901 cells transfected with miR-107 inhibitor or the control. **c**: CDK8 protein level was detected by Western blotting in SGC7901 cells transfected with miR-107 inhibitor or the control. *P < 0.05.

Comparison of luciferase activity in experimental group with negative control group showed that luciferase activity in SGC7901 cells cotransfected with pMIR-REPORT and miR-107 mimics was 38.9% of that in pMIR-REPORT and NC cotransfected group (5.02 ± 2.11 vs. 12.87 ± 6.37, *P* < 0.05, shown in Figure 4a). This data showed that there was specific binding between miR-107 and 3′-UTR in CDK8 gene. CDK8 mRNA expression level was significantly decreased in miR-107 inhibitor transfected SGC7901 cells compared with control group (shown in Figure 4b). Furthermore, CDK8 protein expression measured by Western blotting in miR-107 inhibitor transfected SGC7901 cells was significantly decreased compared with control group (shown in Figure 4c). These results indicated that miR-107 suppressed CDK8 expression posttranscriptionally.

### Down regulation of CDK8 attenuated the oncogenic effect of miR-107

**Figure 5:**
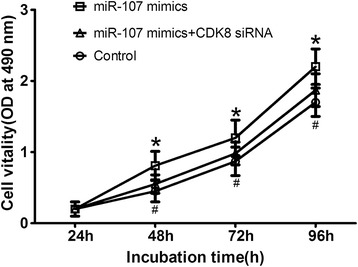
**Down regulation of CDK8 attenuated the oncogenic effect of miR-107.** *P < 0.05 compared with control. #*P* < 0.05 compared with miR-107 mimics group.

Further investigations were performed to study whether down regulation of CDK8 could attenuate the oncogenic effect of miR-107. MTT assay showed that down regulation of CDK8 by siRNA for CDK8 could significantly attenuate the oncogenic effect of miR-107 (shown in Figure 5), suggesting that miR-107 promoted the proliferation of GC cells partially by targeting CDK8.

## Discussion

It is generally accepted that the development of GC, like other cancers, involves multiple steps, including the accumulation of genetic and epigenetic changes. However, the precise mechanism underlying gastric carcinogenesis remains unclear. Therefore, it has been a global research hotspot to looking for new therapeutic targets for GC treatment.

Accumulating evidence has indicated that aberrant expression of miRNAs may be a common mechanism involved in the development of various cancers [3]. Investigation of cancer-specific miRNAs and their targets is necessary for further elucidation of their role in the pathogenesis of tumors, and may be important for the design of novel therapeutic targets [6,8,17]. Although miRNAs have been widely studied in different types of cancers, the knowledge of the aberrant expression and potential function of miRNAs in GC is largely lacking. Accumulating evidence shows that miR-107 is one of the oncogenic RNAs, and overexpression of these RNAs has been reported in several types of human malignant solid tumors. Previously, Inoue et al. found that the mean expression level of miR-107 was significantly higher in the GC tissues compared to that of normal tissues. In the comparison of clinicopathological factors, miR-107 expression showed significant association with depth of tumor invasion, lymph node metastasis and tumor stage. In Kaplan-Meier survival curve analysis, OS and DFS of patients with high miR-107 expression were significantly worse than those of patients with low miR-107 expression. In the Cox multivariate analysis, it was shown that miR-107 expression in GC tissues was an independent prognostic factor for OS and DFS. Their results indicate that miR-107 may be useful as an effective biomarker for prediction of a poor prognosis in GC patients [13]. However, the detailed mechanisms of miR-107 were fewly investigated in GC. Previously, Feng et al. found that miR-107 targeted cyclin-dependent kinase 6 (CDK6) expression, induced cell cycle G1 arrest and inhibited invasion in GC cells [18]. Li et al. found that upregulation of miR-107 induced proliferation in GC cells by targeting the transcription factor FOXO1 [19]. In the present study, we validated that the expression of miR-107 was significantly increased in GC cell line compared with normal controls. Consistent with previous findings from other cancers, such as esophageal cancer, pancreatic cancer and colorectal cancer [9–12], in GC, we also found that miR-107 could remarkably promote cell proliferation and suppress apoptosis. In addition, we found that clone formation rate of miR-107 inhibitor transfected group was significantly lower than that of control group, demonstrating that miR-107 inhibitor significantly inhibited GC cell line colony formation. Thus, our data suggested that miR-107 might play an important role in GC development.

As we know, miRNA functions through interacting with target genes thus the key to explore the mechanism of miRNA is to study the interaction between miRNA and its target genes. In this study, CDK8 was predicted to be the target gene of miR-107 by online biological software, then luciferase reporter vectors containing CDK8 gene 3′-UTR region with miR-107 binding site was constructed and specific binding between miR-107 and CDK8 was verified. The expression level of CDK8 mRNA and protein in miR-107 inhibitor transfected GC cell line was significantly decreased compared with control group,, indicating that miR-107 suppressed CDK8 expression posttranscriptionally. CDK8 is a member of CDK family (CDKs), which is a group of serine-threonine protein kinase and consists of 10 members with different homology. In the past decade, It has been showed that CDKs were excessively activated in different tumors [20]. Preclinical studies have proved that CDKs can promote gene transcription, cell differentiation and angiogenesis [21]. In our study, MTT assay showed that down regulation of CDK8 by siRNA could significantly attenuate the oncogenic effect of miR-107, suggesting that miR-107 promoted the proliferation of GC cells partially by targeting CDK8.

## Conclusion

In conclusion, our findings indicate that miR-107 is upregulated in GC and affects the proliferation of GC cells, partially through the regulation of CDK8. Thus, the identification of the role of miR-107 as an oncogene through targeting CDK8 in GChelps us to further elucidate the potential molecular mechanisms of GC development.
